# Target specific functions of EPL interneurons in olfactory circuits

**DOI:** 10.1038/s41467-019-11354-y

**Published:** 2019-07-29

**Authors:** Gary Liu, Emmanouil Froudarakis, Jay M. Patel, Mikhail Y. Kochukov, Brandon Pekarek, Patrick J. Hunt, Mayuri Patel, Kevin Ung, Chia-Hsuan Fu, Juyeong Jo, Hyun-Kyoung Lee, Andreas S. Tolias, Benjamin R. Arenkiel

**Affiliations:** 1 Program in Developmental Biology, Baylor College of Medicine, Houston, TX 77030 USA; 2 Medical Scientist Training Program, Baylor College of Medicine, Houston, TX 77030 USA; 3 Department of Neuroscience, Baylor College of Medicine, Houston, TX 77030 USA; 4 Center for Neuroscience and Artificial Intelligence, Baylor College of Medicine, Houston, TX 77030 USA; 5 Department of Molecular & Human Genetics, Baylor College of Medicine, Houston, TX 77030 USA; 6 Jan and Dan Duncan Neurological Research Institute at Texas Children’s Hospital, Houston, TX 77030 USA; 7 Department of Pediatrics, Baylor College of Medicine, Houston, TX 77030 USA; 8 Department of Electrical and Computer Engineering, Rice University, Houston, TX 77005 USA

**Keywords:** Neural circuits, Olfactory bulb

## Abstract

Inhibitory interneurons are integral to sensory processing, yet revealing their cell type-specific roles in sensory circuits remains an ongoing focus. To Investigate the mouse olfactory system, we selectively remove GABAergic transmission from a subset of olfactory bulb interneurons, EPL interneurons (EPL-INs), and assay odor responses from their downstream synaptic partners — tufted cells and mitral cells. Using a combination of in vivo electrophysiological and imaging analyses, we find that inactivating this single node of inhibition leads to differential effects in magnitude, reliability, tuning width, and temporal dynamics between the two principal neurons. Furthermore, tufted and not mitral cell responses to odor mixtures become more linearly predictable without EPL-IN inhibition. Our data suggest that olfactory bulb interneurons, through exerting distinct inhibitory functions onto their different synaptic partners, play a significant role in the processing of odor information.

## Abstract (web summary)

The precise cell-type specific role of inhibitory interneurons in regulating sensory responses in the olfactory bulb is not known. Here, the authors report that removing GABAergic inhibition from one layer differentially affects response dynamics of the two main output cell types and changes odor mixture processing.

## Introduction

Understanding the relationship between structure and function in the brain requires investigating neuronal subtypes, mapping their synaptic connections, and evaluating their functional roles in circuit processing. GABAergic interneurons are diverse in their molecular identity, patterns of connectivity, electrophysiological output, and contributions towards sensory processing^1,2^. Recent investigations into the vast diversity of cortical interneurons have led to a fundamental question: To what extent do interneuron subtypes exert distinct effects on their multiple excitatory targets, and if the inhibitory output from one interneuron subtype is removed, are its multiple targets homogenously influenced, or differentially affected?

To address this question, we investigated sensory processing circuits within the mouse olfactory bulb (OB). This system is suited to investigate interneuron target selectivity, given the diverse interneuron population present^3,4^, and that distinct subgroups of OB interneurons can play significant roles, such as gain control^5,6^, in early stages of sensory processing^7^. OB interneurons synapse onto two distinct excitatory projection neurons — tufted cells (TCs) and mitral cells (MCs), which receive direct input from Olfactory sensory neurons (OSNs) and project to cortical targets^8–11^. Since there are no thalamic relays to act as a preprocessing step, inhibition from local interneurons onto TCs/MCs sub serves a critical role in odor information processing^12,13^.

Different types of interneurons contribute to OB circuit functions. Of these, granule cells (GCs) have been central to modeling of olfactory processing^14–16^. GCs represent the most abundant interneuron type in the OB, and are continuously added and replenished via adult neurogenesis^17^. Within the external plexiform layer (EPL) of the OB, EPL-INs are fast-spiking axon-less cells that connect extensively with MCs and TCs^5,18–20^. Interestingly, EPL-INs display higher unitary synaptic strength and connection density onto TCs/MCs than GCs^19^. While each GC connects with up to 200–300 TCs/MCs, individual EPL-INs can synapse with over 1000 TCs/MCs^7^. Thus, such broad and robust patterns of connectivity suggest that EPL-INs perform important odor response computations^7^.

Here, we sought to elucidate three properties of EPL-IN inhibition within the OB: (1) establishing and maintaining OB anatomical circuit architecture, (2) synaptic connectivity of EPL-IN to TCs/MCs, and (3) effects on TC vs MC response properties. Towards this, we combined both in vitro and in vivo techniques that include cell type-specific genetic manipulations, optogenetic mapping, electrophysiology, two-photon imaging, and computational modeling to investigate the roles of EPL-INs.

We find that loss of EPL-IN inhibition leads to single unit electrophysiological changes in TC/MC odor responses, without changing overall OB anatomical organization. Furthermore, EPL-INs more strongly inhibit TCs over MCs, and responses between the two excitatory subtypes differ in magnitude, reliability, tuning width, and temporal dynamics. Interestingly, TC, but not MC, responses to odor mixtures are also more linearly predictable without EPL-IN inhibition. Subsequent modeling suggests that EPL-INs receive inputs from TCs with similar odor responses, and this imparts a form of reciprocal inhibition in a divisive manner. Collectively, our data suggest that EPL-INs exhibit differential inhibitory roles onto distinct excitatory populations, thus shaping target neuron sensory responses.

## Results

### OB anatomical organization is preserved in EPL-IN Vgat knockouts

**Fig. 1:**
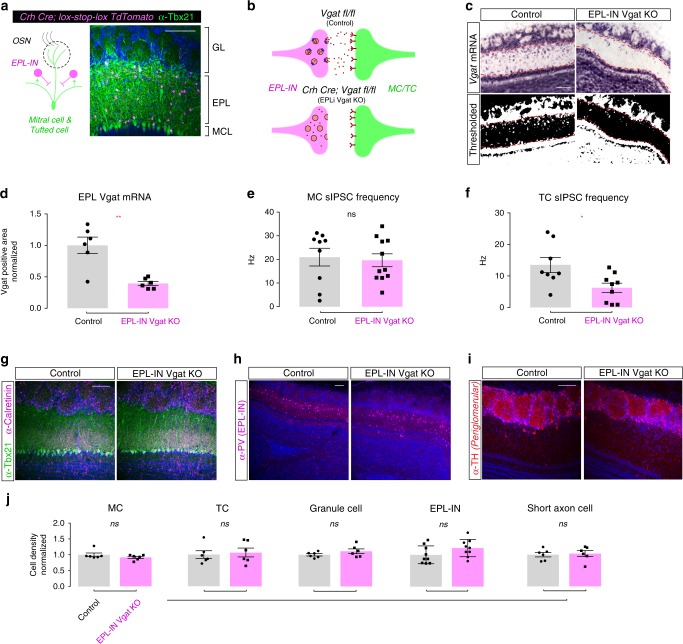
Olfactory bulb architecture is preserved in EPL-IN Vgat knockouts. **a** Illustration of OB connectivity (left). OB coronal section (right) with TdTomato positive EPL-INs (magenta) and anti-Tbx21 positive TC/MC (green) in the EPL and MCL layers. **b** Illustration depicting normal Vgat protein function for neurotransmitter vesicle loading (Top) and after excision of the Vgat^flox/flox^ allele (Bottom). **c** Top: In situ hybridization against VGAT mRNA between control and EPL *Vgat* knockout. Bottom: Thresholded black/white image from top images. **d** Quantification of Vgat positive area within the EPL. All areas normalized to the average control area. Two-tailed *t*-test, **p < 0.01. **e** Frequency measurements of sIPSC onto MCs between Control vs EPL *Vgat* KO. Two-tailed *t*-test. **f** Frequency measurements of sIPSC onto TCs between Control vs EPL *Vgat* KO, Two-tailed *t*-test, *p < 0.05. **g** Antibody staining to compare control and EPL Vgat knockout animals for Tbx21 positive excitatory neurons and calretinin-positive inhibitory neurons, **h** parvalbumin positive EPL-INs, and **i** tyrosine hydroxylase (TH) positive SACs. **j** Cell density comparisons (two-tailed *t*-test) between control and experimental animals for MCs, TCs, GCs, EPL-INs, and SACs. Cell densities normalized to controls. Averages ± SEM. All scale bars 100 μm

To assay the overall function of EPL-IN inhibition onto downstream TCs/MCs, we developed a genetic model for removal of GABA signaling from EPL-INs. Although acute light and ligand-dependent mechanisms for inhibiting neurons are incredibly powerful, they each are limited in the capacity to robustly and/or chronically inhibit neuronal function^21,22^. To circumvent this, we generated mice for conditional deletion of the Vesicular GABA transporter (*Vgat*), which is required for loading GABA into neurotransmitter vesicles. By combining an EPL-IN specific *Crh*-Cre driver^19,23^, and a *Vgat*^*flox/flox*^ allele^24^, we generated conditional mouse models to inhibit GABAergic neurotransmission selectively from OB EPL-INs (Fig. 1a, b).

To confirm targeted deletion of *Vgat* in the EPL, we performed in situ hybridization against *Vgat* mRNA, and visualized transcript changes within the OB (Fig. 1c, top). Whereas strong expression of *Vgat* mRNA appeared normal throughout the granule cell layer, we observed a selective decrease in *Vgat* expression throughout the EPL. To quantify this decrease, we first created black/white thresholded images from in situ hybridization data (Fig. 1c, bottom). We then measured the area of *Vgat* mRNA positive expression within the EPL and observed a significant area decrease in EPL *Vgat* KOs as compared to controls (Fig. 1d, *n* = 6 mice per group, area normalized to controls, average ± SEM, EPL *Vgat* KO: 0.3946 ± 0.034 vs Control: 1.000 ± 0.1317, two-sided *t*-test, *p* = 0.0095).

We also validated that the conditional genetic manipulation impaired release of GABA onto projection neurons by measuring sIPSCs from TCs and MCs. While this revealed that EPL *Vgat* KO does not alter sIPSC frequency onto MCs, we observed a significant decrease in sIPSC frequency onto TCs (Fig. 1e, MC: EPL *Vgat* KO: *n* = 11 cells across 5 mice, 19.62 ± 2.74 Hz vs Control: *n* = 9 cells across 4 mice, 20.96 ± 3.75 Hz, *p* = 0.7702, Fig. 1f, TC: EPL *Vgat* KO: *n* = 9 cells across 5 mice, 6.318 ± 1.50 Hz vs Control: *n* = 8 cells across 4 mice, 13.50 ± 2.41 Hz, *p* = 0.0202).

Next, we investigated the general anatomy of the OB following loss of *Vgat* from EPL-INs. To evaluate the organization of excitatory TCs/MCs and inhibitory interneurons, we stained OB tissue using antibodies against TBX21 and CALRETININ, respectively^25–27^. We did not observe any gross cellular changes between experimental and control animals (Fig. 1g).

These data supported that loss of *Vgat* did not impact the cell number of interneuron subtypes that reside in the EPL. Next, we performed antibody staining using anti-PV, another marker of EPL-INs^18^, and anti-TH antibodies to mark juxtaglomerular cells (JGCs)^28^, respectively (Fig. 1h, i). Again, we did not detect any differences in gross structure or density of OB cell types between experimental and control animals, and thus concluded that EPL-IN inhibition is not necessary for proper anatomical development and the general maintenance of OB anatomy (Fig. 1j, average ± SEM, MCs *n* = 6 OBs in 3 animals, control vs experimental animals, 1.00 ± 0.057 vs 0.91 ± 0.034, ns, *p* = 0.2207, TCs n=6 OBs, 3 animals, 1.00 ± 0.120 vs 1.06 ± 0.141, ns, *p* = 0.7353, GCs *n* = 6 OBs across 3 animals, 1.00 ± 0.037 vs 1.112 ± 0.074, ns, *p* = 0.2035, EPL-INs *n* = 9 OBs across 5 animals, 1.00 ± 0.094 vs 1.21 ± 0.045, ns, *p* = 0.1242, and JGCs n = 6 OBs across 3 animals, 1.00 ± 0.071 vs 1.04 ± 0.095, ns, *p* = 0.7768).

### Loss of EPL-IN inhibition alters odor responses

**Fig. 2:**
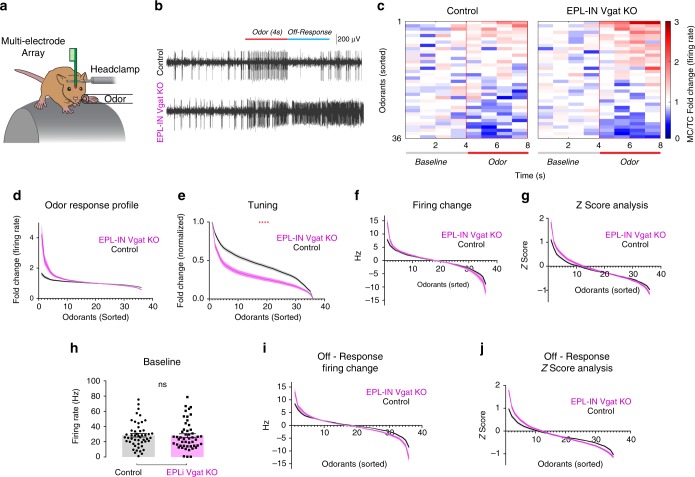
TC/MC odor responses elevate without EPL-IN inhibition. **a** Experimental setup. **b** Representative TC/MC odor response. **c** Representative single cell odor response profile from TC or MC of control and EPL-IN *Vgat* knockout mice. **d** Average TC/MC odor response profiles of control and EPL-IN *Vgat* knockout animals. EPL *Vgat* KO: *n* = 51 cells across 11 mice, Control: n=55 cells across 12 mice, Odor 1*, Odor 36**, *p < 0.05, **p < 0.01, Bonferroni’s multiple comparisons test, *p* < 0.0001, 2-way ANOVA. **e** Normalized tuning curve comparing control vs EPL-IN Vgat knockout, *p* < 0.0001, 2-way ANOVA. **f** Absolute firing change during odor presentation comparing control vs EPL-IN Vgat knockout. Odor 1**, **p < 0.01, Bonferroni’s multiple comparisons test, *p* < 0.0001, 2-way ANOVA. **g** *Z* score analysis of odor response comparing EPL-IN Vgat knockout vs controls. Odor 1**, **p < 0.01, Bonferroni’s multiple comparisons test, *p* < 0.0001, 2-way ANOVA. **h** Baseline frequencies of all recorded neurons, Two-tailed *t*-test. **i** Absolute firing change of Off-Response comparing control vs EPL-IN Vgat knockout, Two-tailed *t*-test, Odor 1****, Odor 35**, Odor 36****p < 0.01, Bonferroni’s multiple comparisons test, *p* < 0.0001, 2-way ANOVA. **j** *Z* score analysis of Off-Response comparing EPL-IN Vgat knockout vs controls. Odor 1****, Odor 2**, **p < 0.01, ****p < 0.0001, Bonferroni’s multiple comparisons test, *p* < 0.0001, 2-way ANOVA. Averages ± SEM

To investigate how EPL-IN inhibition affects odor information processing at a cellular level, we measured TC/MC activity during odor presentation via multi-electrode array recordings in awake, head-fixed animals (Fig. 2a). Electrodes were lowered into the EPL/MC layer based on stereotaxic location^29^, and single units were isolated by spike sorting (see “Methods” section). Next, we presented a panel of 36-odorants (Supplementary Table 1, 2a), and recorded odor-evoked TC/MC responses (Fig. 2b). Because odor responses within the OB can differ by region, we used previously reported odorants that span different activation domains of the OB^9,30–35^. Odor-evoked TC/MC responses were subsequently used to construct normalized response profiles for each control and experimental cell (Fig. 2c). After sorting each profile by odor response, we averaged the profiles for both control and experimental groups.

With loss of EPL-IN inhibition, we observed a significant change in odor response profiles between control and EPL-*Vgat* KO animals (Fig. 2d, EPL *Vgat* KO: *n* = 51 cells across 11 mice, Control: *n* = 55 cells across 12 mice). Furthermore, we observed an increase in firing in response to the most activating odor, with a decrease in firing to the most inhibiting odor. These opposing effects led us to investigate the tuning between the two groups. We first normalized all responses between 0 and 1, relative to the minimum and maximum response for each cell, and then averaged the tuning curves for each group to reveal a significant difference in tuning curve between experimental and control animals (Fig. 2e).

Because fold-change analysis can show inherent biases towards lower baseline firing cells, we also assayed odor responses by absolute firing change and *Z*-score, both of which changed significantly (Fig. 2f, g). Furthermore, TC/MC absolute firing rates were increased in response to the most activating odor (Fig. 2f). *Z*-score analysis also revealed the same pattern of increased odor response (Fig. 2g). We also noted no significant differences in the baseline firing rates between EPL-IN Vgat KO and controls (Fig. 2h, EPL-IN Vgat KO: 28.59 ± 2.34 Hz vs Control: 27.92 ± 2.61 Hz, mean ± SEM, ns, *p* = 0.8484, Two-tailed *t*-test). These data show changes in tuning properties of TC/MC populations with removal of EPL-IN GABAergic neurotransmission.

Blocking inhibition also revealed a marked effect on odorant responses after the odor was removed. To investigate this further, we evaluated the TC/MC firing rates 4 s immediately after odor presentation, which we termed the Off-Response (Fig. 2b, Supplementary Fig. 3). We assayed Off-Responses by comparing absolute firing rate changes (Fig. 2i), which revealed increased responses to the most activating odors decreased responses to the most inhibiting odors. *Z*-score analysis was also significantly changed (Fig. 2j), and revealed an increase in firing rate to the most activating odors. Together, these data show that inhibition from EPL-IN is necessary for maintaining normal odor response profiles.

### EPL-INs provide greater inhibition onto TCs vs MCs

**Fig. 3:**
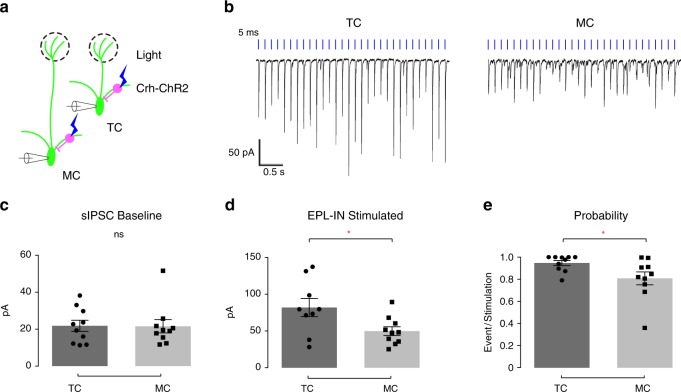
EPL-INs provide stronger inhibition onto TCs than MCs. **a** Illustration depicting targeted voltage clamp recordings of TCs/MCs during optogenetic stimulation of EPL-INs. **b** Representative traces of TCs and MCs during photostimulation. **c** Baseline sIPSC frequencies of TCs and MCs, Two-tailed *t*-test. **d** Light-evoked IPSC amplitude between TCs and MCs, *p < 0.05, Two-tailed *t*-test. **e** Probability of evoked IPSC between TCs/MCs per stimulation. *p < 0.05, Two-tailed *t*-test. *n* = 10 cells/4 animals per group. Average across 30 s ± SEM

MCs and TCs carry distinct odor information^36–38^ and project to different downstream targets^39^. After finding that EPL-IN inhibition is necessary for normal TC/MC odor responses, we sought to determine if EPL-INs differentially regulate TCs and MCs. To assay potential differences at the synaptic level, we compared the level of EPL-IN inhibition received by MCs vs TCs by making whole-cell recordings in acute brain slices and monitoring inhibitory postsynaptic currents. We then photo-stimulated EPL-INs expressing Channelrhodopsin-2 (ChR2), and recorded light-evoked IPSCs in both MCs and TCs (Fig. 3a, b).

Before photo-stimulation, we observed no differences in baseline amplitudes of spontaneous inhibitory postsynaptic currents (sIPSCs) between MCs and TCs (Fig. 3c, *n* = 10 cells across 4 animals, average across 30 s ± SEM, MCs 21.56 ± 3.60 pA vs TCs 21.80 ± 3.00 pA, ns, *p* = 0.9594, Two-tailed *t*-test). However, photo-stimulating EPL-INs revealed a higher peak amplitude of evoked IPSCs onto TCs compared to MCs (Fig. 3d, *n* = 10 cells across 4 animals per group, average across 30 s recording ± SEM, MCs 49.81 ± 6.00 pA vs TCs 81.95 ± 10.95 pA*, *p < 0.05, Two-tailed *t*-test). Furthermore, the probability of eliciting evoked IPSC responses upon light stimulation was greater for TCs than MCs (Fig. 3e, *n* = 10 cells across 4 animals per group, average ± SEM, MCs 0.81 ± 0.059 vs TCs 0.95 ± 0.023*, *p < 0.05, Two-tailed *t*-test). Overall, these data suggest that EPL-INs provide stronger inhibition onto TCs versus MCs.

### EPL-INs exert differential effects onto TCs vs MCs in olfactory processing

**Fig. 4:**
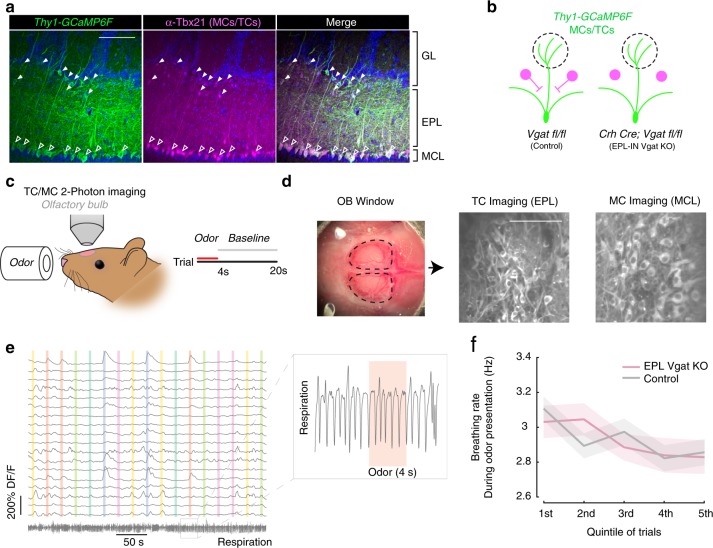
Two-photon imaging of odor responses. **a** *Thy1-GCaMP6F* transgenic mice showing *GCaMP6F* positive MCs (hollow triangle) and TCs (solid triangle) (left) as identified by anti-TBX21 labeling (middle, right). **b** *Thy1-GCaMP6F* allele in control and EPL-IN *Vgat* knockout animals. **c** In vivo 2-photon imaging in awake, head-fixed rodents. **d** Example cortical window for 2-photon experiment (left). Example GCaMP imaging of different depths for TCs (middle) and MCs (right). Scale bars, 100 μm. **e** Example calcium traces from 18 TCs, with odor stimulation periods represented as colored bars. Scale bars represent 200% df/f. The respiration measured as temperature modulation is shown in the bottom trace. Insert shows a small segment of the respiration during one trial. **f** Average breathing rate (Hz) as a function of time in the experiment for all animals

Having revealed differences in strength of EPL-IN inhibition onto TCs vs MCs, we next sought to identify how EPL-INs may differentially affect odor responses between the two cell types. Towards this, we introduced a *Thy1-GCaMP6F* reporter^40^ into both control *Vgat*^*flox/flox*^, and *Crh*-Cre; *Vgat*^*flox/flox*^ experimental animals. To evaluate if *GCaMP6F* expression was restricted to TCs/MCs, we performed antibody staining against *Tbx21*. Consistent with genetically restricted cell type-specific expression in TCs/MCs of the OB, the *Thy1-GCaMP6f* reporter showed strong overlap with *Tbx21* positive cells (Fig. 4a, b).

MCs and TCs are located within different layers of the OB and we used in vivo 2-photon imaging in awake animals to study their properties^41,42^. By recording at different depths, we measured the activity of TC bodies vs MC bodies^42^ in both EPL-IN *Vgat* KO and control animals (Fig. 4c, d). We presented 5 repetitions of 2 different odor panels, each consisting of both single odorants and 2-odor mixtures, at a concentration of 100 parts per billion (ppb) for each odor (28 total odors/panel, see “Methods” section) (Supplementary Table 2b) (*n* = 7 EPL *Vgat* KO and 8 Control animals). To measure odor responses, we identified and manually segmented cell bodies (Supplementary Fig. 1) to quantify their ΔF/F responses (Fig. 4e).

Previous studies have shown that odor responses vary with changes in respiration^43^. Therefore, we first measured respiration rates throughout recording sessions to determine if changes in breathing patterns differed between control and EPL *Vgat* KO animals. Using an external thermocouple (Supplementary Fig. 2), we quantified respiration rate throughout odor presentation. In agreement with previous reports, the average respiration rate of control animals was about 3 Hz^44^, and we detected no differences in respiration rates between experimental and control groups (Fig. 4f).

**Fig. 5:**
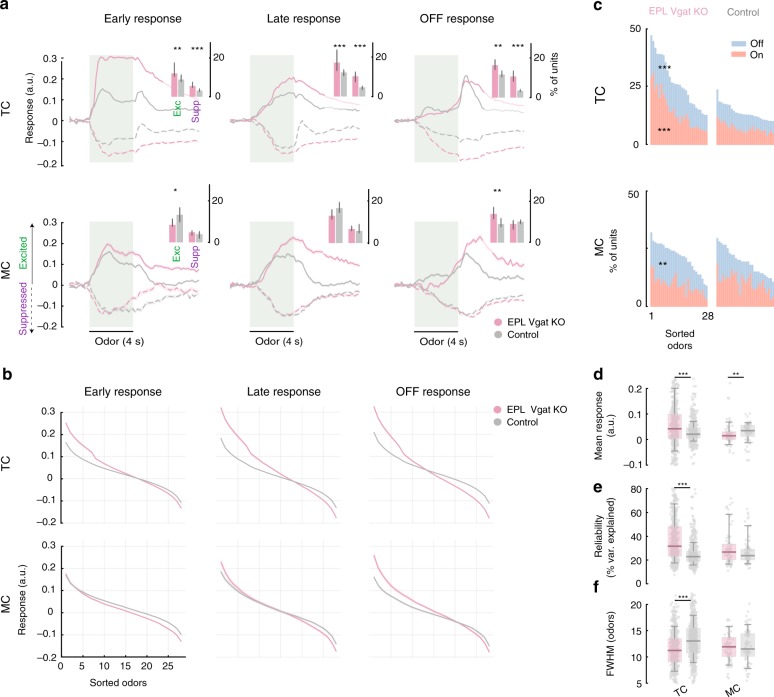
TC and MC odor responses are differentially affected. **a** Average calcium responses to odors across all significant cell-odor pairs separated by period of a significant response (uniform line) and suppressed (dotted line) units between EPL *Vgat* KO and control animals. TC (top), and MC (bottom). Insert bar plot shows the percentage of responses significantly altered. Responses divided by response latency into Early (first 2 s of odor), Late (last 2s of odor), and Off (2s after odor is turned off). The number of unit-odor pairs: excited KO group: Early: TC = 5331, MC = 480 Late: TC = 2688, MC = 395, OFF: TC = 1910, MC = 246 suppressed KO group: Early: TC = 2396, MC = 290 Late: TC = 2711, MC = 361, OFF: TC = 4011, MC = 502 excited Control group: Early: TC = 6679, MC = 566 Late: TC = 4650, MC = 305, OFF: TC = 4879, MC = 181 suppressed Control group: Early: TC = 2229, MC = 153 Late: TC = 3057, MC = 295, OFF: TC = 2557, MC = 478. **b** Average tuning functions across all cells with significant responses. Shaded area represents SEM computed over cells. **c** Histograms represent the total fraction of cells with significant odor responses. Red is significant for on response (Early), blue is significant for off response. **d** Average response strength across all odors. **e** Reliability of odor response (see “Methods” section). **f** Tuning width as the FWHM of the fitted exponential function for each cell as in (**d**). For **d**–**f** Box plots are computed across all cells — bounds of the box span from 25 to 75% percentile, center line represents median, and whiskers visualize 10 and 90% of the data points. For visibility, the extreme outliers are not presented. The number of cells for EPL *Vgat* KO animals: TC = 646, MC = 66, for control animals: TC = 808, MC = 72, ***p < 0.001, **p < 0.01, *p < 0.05, Wilcoxon rank sum test

We observed striking differences in temporal kinetics and magnitude of response after loss of EPL-IN inhibition between TCs and MCs. Notably, TCs displayed stronger odor responses compared to those observed for MCs (Fig. 5a–d, Supplementary Fig. 4). Furthermore, after loss of the inhibition, TCs but not MCs, exhibited longer times to reach peak activity (Supplementary Fig. 5). To better analyze these differences, we subdivided odor responses based on latency into early (first 2s of odor), late (last 2s of odor), and off (2 s after odor delivery) time periods. Following loss of EPL-IN inhibition, amplitudes of TC responses increased, which was reflected in both the averaged traces and the tuning profiles (Fig. 5a, b). In KO animals, changes in amplitude of MC response occurred more during the late and off phase than during the initial phase. Overall, mean responses across all odor-cell pairs was increased in TCs and decreased in MCs (Fig. 5d, median %df/f and 90% upper/lower confidence interval, experimental vs control, TC 4.2, 3.7/4.6 vs 2.1, 2/2.3 *p* < 0.001, MC 1.5, 0.9/2 vs 3.5, 2.9/4.2 *p* < 0.01, Wilcoxon rank sum test).

Interestingly, the percentage of TCs that responded to odors increased at each phase of odor presentation (Fig. 5a bar plot: %units and 90% upper/lower confidence interval, experimental vs control, Early excited: TC 12.1, 10.7/16.3 vs 8.9, 8.1/10.6, MC 8.4, 7.8/10.8 vs 13.3, 10.1/16.5, Early suppressed: TC 5.5, 5/6.9 vs 3.2, 2.9/3.9, MC 4.8, 3.6/5.1 vs 4, 2.5/5, Late excited: TC 17.2, 13.3/22.3 vs 12.2, 10.7/13.1, MC 13, 11.4/15.7 vs 16.5, 14.7/19.1, Late suppressed: TC 10.3, 7.7/12.2 vs 4.6, 3.8/5.1, MC 6.6, 6/7.8 vs 5.8, 5.4/7.9, OFF excited: TC 16.3, 14.4/19.1 vs 11.6, 10.6/13.2, MC 13.9, 11.7/15.7 vs 9, 7.9/11.5, OFF suppressed: TC 10.7, 8.4/13.2 vs 3.4, 3.3/3.9, MC 9, 6.6/10.5 vs 10.1, 9/10.8, *p < 0.05, **p < 0.01, ***p < 0.001, *n* = 28 odors, Wilcoxon rank sum test, Fig. 5c, Supplementary Fig. 6). Both odor-evoked excitation and suppression increased, whereas percentages of unresponsive TCs decreased. Unlike TC responses, the proportion of MCs impacted by the loss of EPL-IN GABA signaling was much less dramatic, showing a decrease in percentage of excitatory MC responses during early periods of odor delivery, and increased responsivity during the off response (Fig. 5a–c, Supplementary Fig. 2, 5). Unlike TCs, the percentage of unresponsive MCs was not significantly altered.

Other measures of neural activity were differentially affected as well. Reliability, which estimates the consistency of responses across trials, was increased after removing EPL-IN inhibition in TCs, but remained unchanged in MCs (Fig. 5e, median, U/L 90% CI experimental vs control, TC 31.6%, 30.6/32.5% vs 22.9%, 22.5/23.2% *p* < 0.001, MC 26.8%, 23.9/29.2% vs 23.8%, 22.1/26, Wilcoxon rank sum test). Moreover, the selectivity of neuronal responses to the odorants was higher in KO animals. To further evaluate this, we estimated a neuron’s odor tuning as the full width half max (FWHM) of the exponential fit to the sorted odor responses for each odor. After removing EPL-IN inhibition, tuning was narrowed in TCs, but was unchanged in MCs (Fig. 5f, median, U/L 90% CI, KO TCs: 11.3, 11/11.4 vs Control TCs: 13, 12.8/13.2 *p* < 0.001, KO MCs: 11.9, 11/12.7 vs Control MCs: 11.5, 11/12.5, Wilcoxon rank sum test). Together, these findings suggest that loss of EPL-IN inhibition more strongly impacts TC odor responses, and that EPL-INs exhibit preferential and sometimes opposing effects on specific parameters of TC and MC olfactory responses.

### Cortical projecting TC responses are altered in EPL-IN *Vgat* KO animals

**Fig. 6:**
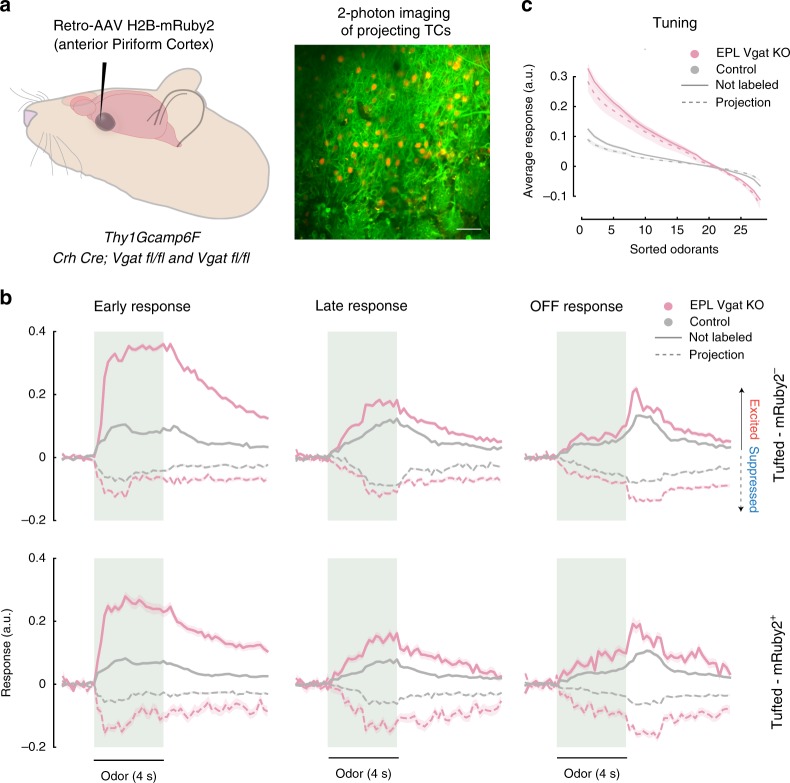
Projecting TC and general TC responses are similarly affected without EPL-IN. **a** Experimental setup of Retro-AAV *H2B-mRuby2* viral injection into the anterior piriform cortex (left) and subsequent 2-photon imaging 2 weeks following injection (right). **b** Calcium responses to odors across all significant cell-odor pairs for excited (uniform line) and suppressed (dotted line) units between EPL *Vgat* KO and control animals. Responses divided into Early (first 2s of odor), Late (last 2s of odor), and Off (2s after odor is turned off). mRuby2 negative TC (top) and mRuby2 positive TC (bottom). The number of unit-odor pairs: excited KO group: Early: mRuby2− = 2149, mRuby2+ = 394 Late: mRuby2− = 881, mRuby2+ = 168, OFF: mRuby2− = 563, mRuby2+ = 127, suppressed KO group: Early: mRuby2− = 524, mRuby2+ = 103 Late: mRuby2− = 425, mRuby2+ = 101, OFF: mRuby2− = 761, mRuby2+ = 191, excited Control group: Early: mRuby2− = 1650, mRuby2+ = 1138 Late: mRuby2− = 1375, mRuby2+ = 876, OFF: mRuby2− = 1261, mRuby2+ = 517, suppressed Control group: Early: mRuby2− = 470, mRuby2+ = 319 Late: mRuby2− = 705, mRuby2+ = 326, OFF: mRuby2− = 717, mRuby2+ = 515. **c** Average tuning functions across the population. Shaded area represents SEM computed over unit response. The number of cells for EPL *Vgat* KO animals: mRuby2− = 240, mRuby2+ = 44, for control animals: mRuby2− = 154, mRuby2+ = 126

TCs can be divided into different subtypes^3^, with External TCs synapsing locally within the OB, and Middle and Internal TCs with projections to the olfactory cortex. To investigate how EPL-INs regulate projecting TCs (pTCs) vs MCs, we identified and isolated pTCs and recorded their responses under control vs experimental conditions. Towards this, we selectively labeled pTCs in the OB by first injecting a Retro-AAV-H2B-mRuby2 virus^45^ into the ventrorostral anterior Piriform Cortex (Fig. 6a) where pTCs send robust projections^39^, and then imaged odor responses in mRuby2 expressing pTCs within the EPL.

Much like the responses of general TCs, pTCs were similarly impacted by loss of EPL-IN GABAergic neurotransmission (Fig. 6b). As observed in general TC/MC populations, odor responses in early, late, and off phases were altered in EPL-IN Vgat KO animals. Upon removing EPL-IN inhibition, both mRuby2+ and mRuby2− TCs displayed increased magnitudes of excited and suppressed odor responses. Furthermore, tuning was also similarly affected, where both mRuby2+ and mRuby2− TCs displayed higher responses to the most excitatory odors, and lower responses to the most inhibitory odors (Fig. 6c). Thus, pTCs were functionally impacted by loss of EPL-IN Vgat in a similar manner to TCs lacking the mRuby2 reporter.

### EPL-INs influence TC responses to odor mixtures

**Fig. 7:**
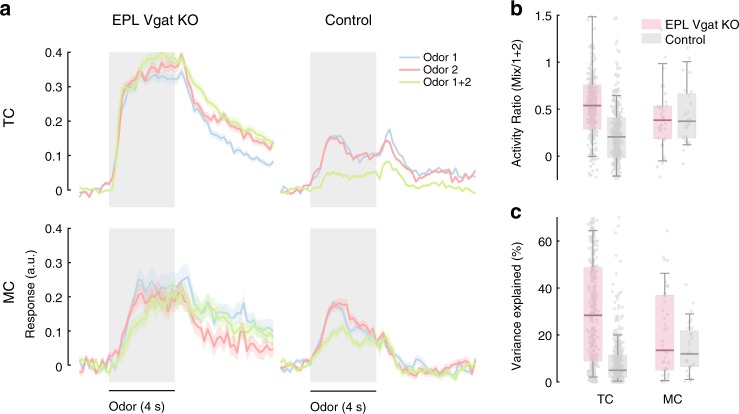
Responses to odor mixtures become more linearly predictable without EPL-IN. **a** Average TC and MC calcium responses from experimental and control animals to 2 odorants and their mixtures. Error bands indicate SEM. **b** Ratios between the response to odor mixtures, and the sum of the responses to the constituent odorants. Responses represent the average response profile 6s (includes both ON and OFF responses) from odorant onset. **c** % Variance explained of a linear regression model built to predict the responses to odor mixtures from the responses to single odors. For **b**, **c** Box plots are computed across all cell-odor pairs — bounds of the box span from 25 to 75% percentile, center line represents median, and whiskers visualize 10 and 90% of the data points. The number of cells for EPL *Vgat* KO animals: TC = 357, MC = 41, for control animals: TC = 407, MC = 28. ***p < 0.001, Wilcoxon rank sum test

In the natural environment, odors are inhaled as mixtures that elicit complex TC/MC responses. While general rules do exist, predicting mixture responses from individual odor constituents remains a challenge^42,46^. The complex and ‘non-linear’ aspect of mixture coding has been previously attributed to inhibition^1^. To determine if EPL-INs contribute to non-linear coding of odor mixtures, we presented 2 different odor panels as both single odors and pairwise odor combinations. We selected pairs of odors that produced significant responses when presented in isolation, and then compared combined mixture responses to the sum of the individual odor responses (Fig. 7a).

We found that responses to odor mixtures as a linear sum of the individual odor responses affected TCs and MCs differently following EPL-IN *Vgat* KO. TCs exhibited more linearly additive responses to mixtures in KO animals, while MC responses remained unchanged compared to controls (Fig. 7b, median, U/L 90% CI, experimental: TC 0.54, 0.51/0.58, MC 0.38, 0.3/0.45, control: TC 0.21, 0.18/0.22, MC 0.37, 0.31/0.46, *p* < 0.001 Wilcoxon rank sum test). We sought to explain these discrepancies by testing a linear regression model to predict the mixture responses against individual odors. When EPL-IN inhibition was removed, a linear model better predicted the variance of TC mixture responses, indicating that EPL-INs introduces a non-linear inhibitory component to TC output (Fig. 7c, median, U/L 90% CI, experimental: TC 28.5, 23/31, MC 13.6, 8.5/24.5, control: TC 5.2, 4.6/6.1, MC 12.1, 7.7/18.3, *p* < 0.001 Wilcoxon rank sum test).

### Modeling EPL-IN coding of odor mixtures

**Fig. 8:**
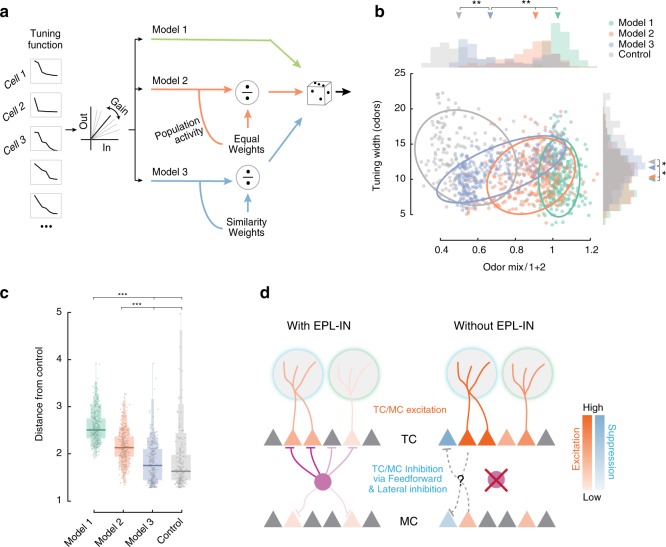
Model of the TC responses. **a** Schematic of three LNP models used to generate the simulated responses (see “Methods” section). **b** Scatter plot of tuning width as in Fig. 5f (right histogram with arrows representing medians) and activity ratio of odor mixtures as in Fig. 7b (top histogram with arrows representing medians), for the three models compared to the tuning width of the TCs in Control animals (gray). *n* = 300 for all three models and *n* = 199 for control data. Ellipses represent the 80^th^ percentile confidence intervals. Note that for visibility purposes only data within 3 standard deviations from the median are included in the scatterplot. *p < 10^–5^, **p < 10^–18^, one-sided two-sample Kolmogorov–Smirnov test. **c** Average mahalanobis distances in (**b**) between each of the cells of model/control performance and the control data. Lines indicate the median of the distribution, and the variable bars indicate the 75, 90, 95, and 100 percentiles. Dots represent the d’ for each unit (*n* = 300 for all three models). ***p < 10^–24^, Wilcoxon rank sum test. **d** Schematic of the proposed connectivity and function of EPL-INs in the olfactory bulb. Model shows preferential changes in TC odor responses upon genetic silencing of EPL-IN

By what mechanism do the EPL-INs introduce this non-linearity to mixture coding? Considering previous work, we tested three different linear-nonlinear Poisson (LNP) models whereby EPL-INs could exert a distinct non-linear effect on TC odor responses. We trained each model using population data of TC responses to single odorants from control and experimental animals. As a linear input to all three models, we used the tuning functions from the significantly tuned TCs measured in EPL-IN *Vgat* KO animals. We then attempted to match TC tuning functions in control animals (see “Methods” section). To determine validity, we compared how accurately each model reflected the differences in tuning width and mixture responses between experimental and control animals. The non-linearity of each model was varied in the following manner: The first model represented a basic gain control model (Fig. 8a), based on data in which MC responses to single odorants were recorded while manipulating EPL-IN activity^5^. In this model, EPL-INs were posed to modulate the magnitude of response for each cell-odor pair with no interactions across neurons.

In the second model, the effect of EPL-INs onto the TCs was designated to reflect the activity of neighboring TCs in a divisive manner, whereby each neuron’s activity is divided by the sum of a weighted population activity, a process first characterized in drosophila olfaction^47,48^. In this divisive normalization model, inhibition is non-specific, and the weights of the population responses contribute to the firing of each neuron equally for each TC (Fig. 8a).

In the third model, we included a divisive normalization model, whereby EPL-IN inhibition was specific, and the weights reflected the tuning similarities between nearby neurons (Fig. 8a). As such, the final model assigned the weights of the inhibitory outputs depending on the response similarities between both EPL-INs and TCs. This third model accounts for regional odor response similarities, a phenomenon first demonstrated by odor mapping, which showed that neurons with similar response profiles are more likely to be located closer to each other^33^, which we also observe (Supplementary Fig. 7).

While all three models could adequately fit the measured odor responses of TCs in control animals, using weights that reflected regional similarities in odor responsivity (Model 3) provided a significantly better fit (Supplementary Fig. 8a). The resulting tuning widths from Model 3 were significantly larger than the tuning widths of Models 1 and 2, but still smaller than those observed in control data (Fig. 8b, median, U/L 90% CI, Model 1: 10.1, 9.7/10.5, Model 2: 10.2, 9.7/10.5, TCs: 12.4, 12.1/12.8 vs Model 3: 11.8, 11.4/12, *p < 10^–5^ one-sided two-sample Kolmogorov–Smirnov test). In a similar manner, responses to mixtures were closer to the linear sum of the responses to individual odors for Models 1 and 2 when compared to the responses of Model 3. (Fig. 8b, median, U/L 90% CI, Model 1: 1.03, 1.02/1.04, Model 2: 0.90, 0.88/0.91 vs Model 3: 0.67, 0.64/0.71, **p < 10^–18^, one-sided two-sample Kolmogorov–Smirnov test). Model 3 was closer to the control data but still significantly higher (Fig. 8b, median, U/L 90% CI, TCs: 0.5, 0.49/0.53 vs Model 3: 0.67, 0.64/0.71, **p < 10^–18^, one-sided two-sample Kolmogorov–Smirnov test).

To quantify how well the three independent models predicted tuning widths and responses to odor mixtures of control animals, we evaluated cluster separation in a scatter plot of Fig. 8b as the average Mahalanobis distance of each of the models and control cells to the control data. The performances of both Models 1 and 2 were significantly worse than that of Model 3 at predicting the responses of neurons from control animals. Notably, predicted responses of Model 3 were not distinguishable from recorded control data (Fig. 8c, median, U/L 90% CI, Model 1: 2.51, 2.48/2.54, Model 2: 2.14, 2.10/2.17 vs Model 3: 1.76, 1.67/1.83 and TCs: 1.64, 1.58/1.67). Thus, the best fit model predicts that TCs receive more inputs from EPL-INs with similar tuning, which would imply that co-tuned TCs are inhibited by EPL-INs in a divisive manner.

## Discussion

Our in vivo electrophysiological data reveal that loss of EPL-IN inhibition elevates the magnitude of odor responses within projection neurons of the OB. Whole-cell recordings showed that EPL-INs inhibit TCs more strongly and reliably than MCs, and in vivo 2-photon imaging revealed that loss of EPL-IN inhibition imparts differential effects on MC and TC odor responsivity, including changes in magnitude, reliability, tuning width, and temporal dynamics between the two cell types. TC, but not MC responsivity became more linearly predictable to odor mixtures without EPL-IN inhibition. Further, modeling suggested that EPL-INs receive inputs from TCs with similar tuning, which ultimately functions to inhibit TCs in a divisive manner (Fig. 8d).

Notably, we found that TC responses to odor mixtures were significantly altered after the loss of inhibition from EPL-INs (Fig. 7). Odor mixtures coding offers a unique challenge to the brain, in that odor space is highly dimensional in its possible combinations and concentrations. One way in which mammals may encode this magnitude of information is by responding to each mixture as a non-linear combination of each individual constituent. This may impart more diversity than simply adding or averaging individual odorant responses. Thus, by diversifying the responses of TCs, EPL-INs may serve to better separate the coding of similar odor mixtures.

To better understand EPL-IN function, we tried to use models that reflected both the circuitry within the OB, while also implementing our experimental and control data. The first in vivo characterizations of EPL-IN resulted in a gain control model (Model 1), which is most typically described for feedback inhibition. However, this gain control model did not account for the changes in tuning we observed by both electrophysiological recordings and 2-photon imaging. Thus, we also generated divisive normalization models (Model 2, 3), a process observed in the primary visual cortex, retina, thalamus, and the auditory cortex^47,48^. By normalizing odor-driven activity of TCs by the weighted average activity of surrounding excitatory neurons, divisive normalization allows for EPL-INs to simultaneously influence different features of sensory processing, such as gain and tuning. Furthermore, divisive normalization was originally proposed to explain non-linear responses in the primary visual cortex^48^, which has since been implicated to also function in olfaction.

If coding of odor mixtures is critical towards olfaction, then why are MCs excluded from this computational aspect of EPL-INs? One consideration may be that another interneuron population is responsible for such a task since TCs and MCs have differential processing and routing of odor information^39^. For example, GCs are fundamental in all models of olfactory processing^49,50^. Given that GCs greatly outnumber any other interneuron subtype in the OB, they certainly may influence MC non-linearity.

Glomerular-layer interneurons are also another likely candidate to affect MC non-linearity, as they preferentially inhibit MCs over TCs^51^. For example, Periglomerular Cells (PGCs) are a morphologically and functionally heterogeneous cell population with cell bodies located in the juxtaglomerular space. Previous studies have revealed these neurons as regulators of baseline and odor-evoked inhibition of MCs^52^. Furthermore, PGCs preferentially target MCs over TCs, leading to delays in MC firing per respiration cycle^52^.

We found that loss of GABAergic drive from EPL-INs resulted in opposing effects on the intensity of odor responses by MCs and TCs during the first 2s of odor presentation. One possible explanation for this could include feedforward properties that emerge from the intraglomerular network, which may connect TCs to MCs. Previous studies suggest that MCs are more strongly influenced by glomerular layer inhibition than TCs^36,51^ — this may result from TC activation triggering feedforward inhibition onto MCs. Another example could be lateral inhibitory networks that connect MCs and TCs to neighboring MCs and TCs. These lateral interactions may underlie the increased inhibitory responses observed from MCs and TCs (Fig. 8d).

One potential weakness in this study is that we embryonically removed *Vgat* in all Crh neurons of the brain, leaving open the possibility that non-OB sources of inhibition may be contributing to our phenotypes. Previous studies that have mapped the locations of Crh neurons in the mouse brain, show that regions with projections into the OB, like the piriform cortex, contain Crh neurons with small soma sizes, consistent with local interneurons^53^. Therefore, we cannot rule out the possibility of a polysynaptic mechanism whereby disabling inhibition within regions like the piriform cortex may also contribute to our observations. However, the proportion of Crh neurons in the OB is much larger when compared to the cortex. While cortical interneurons are greatly outnumbered by projection neurons^54^, Crh neuron in the OB are equal in number to all OB projection neurons combined^7^. Furthermore, cortical interneurons are incredibly heterogeneous^2^, which further dilutes the proportion of Crh neurons in the cortex that may directly influence local OB processing. Thus, the much higher proportion of Crh neurons in the OB, coupled with the dense reciprocal connectivity observed onto MCs/TCs suggests that OB Crh neurons are a much more plausible source of inhibition underlying our experimental and modeling observations.

The difference in regulation of MCs vs TCs by EPL-INs has some broad implications for information processing by interneurons. Collectively, our data suggest that EPL-INs can perform unique computational tasks for different subsets of their synaptic partners. Because excitatory and inhibitory cells often form dense and spatially broad connections, this form of task compartmentalization would allow for single interneurons to participate in diverse functional roles. For example, MCs and TCs are often described as parallel output tracts^37,39^, as they both receive olfactory information from OSNs. However, these output neurons encode olfactory information differently. Some of these differences may be due to intrinsic variabilities, such as molecular identity, anatomy, and electrophysiology^32,39^. Our data suggest, however, that external regulation from EPL-INs may also contribute to those differences. Because MCs and TCs project to non-overlapping brain regions^39^, how they differentially encode odor information may indeed affect how various features of olfactory information are relayed to downstream targets.

Molecular and wiring profiles have increasingly revealed the heterogeneity of neurons, yet we still know very little about the functional necessity of given subclasses. By using cell-type specific genetics, our study reveals that the loss of neurotransmission from a single interneuron subtype within the OB can lead to clear alterations in olfactory responses. These alterations were found more often in TCs than MCs, implying a selective deployment of information processing. This suggests that a neuron can connect to many partners, yet primarily influence a subset of them. The target choice may be dependent on many factors, such as synaptic strength, or the activity of other connecting neurons. Thus, changes in any of these factors may even shift the primary target from one neuron to another, creating a dynamic environment to facilitate information processing, and ultimately sensory perception.

## Methods

### Experimental mouse lines

All procedures performed on mice were carried out in accordance with the ethical guidelines of the National Institutes of Health and approved by the institutional review board (IACUC Baylor College of Medicine). *Crh*-Cre^+/−^ (Crh^tm1(cre)Zjh^), *Vgat*^*flox*^ (Slc32a1^tm1Lowl^), *ROSA26 Lox-stop-Lox ChR2-EYFP* (GT(ROSA)26Sor^tm32.1(CAG−COP4*H134R/EYFP)Hze/J^), and *Thy1-GCaMP6f* (Tg(Thy1-GCaMP6f)GP5.11Dkim)) mice were obtained from the Jackson Laboratories.

### Immunohistochemistry, imaging, and cell density analysis

Mice were anesthetized using isoflurane, then sequentially intracardially perfused with PBS, followed by 4% PFA. Intact brains were extracted, fixed in 4% PFA at 1 h room temperature, cryoprotected in 30% sucrose solution for 24 h, and frozen in embedding medium (OCT compound, Fisher). 50 μm slices were cryosectioned (CM1860, Leica) and incubated in blocking solution (10% normal goat serum, 0.3% Triton X-100) at room temperature for 1 h. Sections were stained using the following antibodies diluted in blocking solution overnight at 4 °C: mouse anti-Calretinin 1:1500 (MAB1568, Chemicon), mouse anti-Parvalbumin 1:3000 (MAB1572, Millipore), rabbit anti-Tyrosine Hydroxylase 1:2000 (Ab152, Chemicon), or rabbit anti-Tbx21 1:500 (gift from Mitsui Lab). Sections were then washed 4X, 10 min each in 0.1% Triton X-100 in PBS at room temperature, then incubated in secondary anti-rabbit or mouse conjugated antibodies 1:500 for 1 h at room temperature (Alexa-488nm and Alexa-555nm, Invitrogen). Slices were washed 4X, 15 min each, and mounted with DAPI containing mounting medium (Vectashield, Vector Laboratories). Images were collected using a confocal microscope (TCS SPE, Leica). Neuronal cell densities were quantified by analyzing 3 different 250 μm^2^ fields of view per olfactory bulb.

### Probe generation and in situ hybridization

Vgat sense and antisense probes were generated by PCR amplifying OB cDNA using the following primers- Forward: TGACAGAATTCGCCATTCAGGGCATGTTC and Reverse: ATGGCAAGCTTAGCAGCGTGAAGACCACC. The resulting product was digested with EcoRI (R0101S, NEB) and HindIII (R0104S, NEB), and cloned into SLAX 12 NCO vector (gift from HK Lee lab). The resulting plasmid was confirmed by sequencing, then linearized and transcribed using a DIG RNA labeling mix (11277073910, Roche) in the forward or reverse orientation to generate the sense or antisense probes, respectively. In situ hybridization was then performed on adult mice olfactory bulbs. Adult mice olfactory bulbs were fixed in 4% paraformaldehyde and then cryoprotected using 20% sucrose overnight. Tissues were then sectioned, rinsed in PBS, and treated with Proteinase K (Roche) diluted to 1:200 for 5 min at room temperature. Following re-fixation for 15 min with 4% paraformaldehyde, sections were incubated in standard hybridization buffer solution for at least 1 h at 65 degrees Celsius. Sections were then incubated in RNA probe mixed in hybridization buffer overnight at 65 degrees Celsius. The next day, sections are washed in standard saline-sodium citrate buffer at 65 degrees Celsius, then incubated first in blocking solution (PBT + 20% goat serum) and then diluted in anti-DIG alkaline phosphatase antibody (Sigma) diluted 1:2000 in blocking solution. Slides were then developed until the desired signal using a BCIP/NBT solution (Sigma). Following image acquisition of developed slides, black/white thresholded images were then derived using custom Matlab script and the area of staining within the EPL was obtained using Fiji software.

### Slice preparation and recordings

Animals were deeply anesthetized with isoflurane and perfused intracardially with ice-cold artificial CSF (ACSF) containing the following (in mM): 122 NaCl, 3 KCl, 1.2 NaH_2_PO_4_, 26 NaHCO_3_, 20 glucose, 2 CaCl_2_, and 1 MgCl_2_ at 305–310 mOsm, pH 7.3. Brains were dissected, embedded in low-melting-point agarose, sectioned to 300 μM on a microtome (VT1200, Leica), and placed in ice-cold oxygenated (5% CO_2_, 95% O_2_) dissection buffer containing the following (in mM): 87 NaCl, 2.5 KCl, 1.6 NaH_2_PO_4_, 25 NaHCO3, 75 sucrose, 10 glucose, 1.3 ascorbic acid, 0.5 CaCl_2_, and 4 MgCl_2_. Sections were recovered (at least 30 min at 37 °C) in oxygenated ACSF, then acclimated at room temperature for another 30 min before recordings. Slices were placed in a recording chamber on a fixed stage of an upright microscope (SliceScope Pro 6000 platform, Scientifica) equipped with optiMOS camera (QImaging), and perfused with oxygenated ACSF at room temperature. MCs or TCs were identified using DIC imaging by characteristic morphology and location in the bulb (MCs were within the MCL and TCs were within the EPL), and further confirmed based on membrane properties (large cells with capacitance > 50 pF and membrane resistance < 200 MΩ). Fluorescent imaging was performed with a CoolLED pE-100 470 nm (EGFP) excitation light source. Whole-cell voltage- clamp recordings were made using a Multiclamp 700B amplifier (Axon CNS, Molecular Devices, Sunnyvale, CA, USA) at room temperature (25–27 °C). Electrodes were prepared from borosilicate glass (outer diameter 1.5 mm) using a micropipette puller (Sutter Instruments), pulled to tip resistances between 3–6 MΩ, and filled with high-Cl internal solution, containing the following (in mM): 50 CsMeSO_3_, 50 CsCl, 15 TEA-Cl, 1 MgCl_2_, 0.2 EGTA, 15 HEPES, 4 Na_2_-ATP, 0.3 Na-GTP, 5 QX-314 Chloride, and 14 creatine phosphate, 300–310 mOsm, 14 pH 7.3. Spontaneous and evoked inhibitory postsynaptic currents were recorded in oxygenated ASCF with the addition of 10 μM CNQX and 20 μM D-AP5 to block glutamatergic inward currents. Neurons were held at −70 mV throughout the experiment. The recorded current was digitized at 20 KHz using a Digidata 1440A and Clampex 10.6.2.2 software (Molecular Devices), and stored on a PC hard disk. After prerecording spontaneous inhibitory currents (sIPSC) for 30s, slices were illuminated with 5 ms blue light pulses at 10 Hz for 30s, using CoolLED pE-100 470 nm light source, triggered by TTL signal from Clampex software to evoke IPSC by exciting ChR2 expressing EPL-IN. Off-line analysis included filtering at 1 kHz, and was performed using MiniAnalysis (Synaptosoft Inc, Fort Lee, NJ) and Clampfit 10.7.0.3 (Molecular Devices) software.

### Animal preparation for in vivo electrophysiology recordings

Both adult male and female *Crh*-Cre; *Vgat*^*flox/flox*^ and *Vgat*^*flox/flox*^ mice were used for in vivo recordings (10 and 10 mice, respectively, age: 8+ weeks). Mice were anesthetized with 1–4% isoflurane, and a small incision was made over the middle of the cerebral cortex and extended towards the cerebellum. Small sterilized anchoring screws (3 mm in length) were implanted into the skull. Sterilized custom-made aluminum head plates (0.5 mm thick, 1 cm long, 0.5 cm wide) were fixed to the skull surface using acrylic dental cement (C&B Metabond, Parkell). The plate was further secured to the scalp with tissue adhesive (VetBond, 3M) and the skin sutured. Any skin opening was sealed with silicone elastomer (Kwik-Cast, WPI). Animals were allowed to recover for 1 week after surgery. Prior to recordings and under anesthesia with 1% isoflurane, a craniotomy was made over the dorsal surface of the olfactory bulb. The animals were then transferred to cylindrical treadmill and given at least 3 h before the start of recording to acclimate to head fixation and to recover from the isoflurane.

### In vivo electrophysiology recordings and spike sorting

Single unit recordings from M/T cells were isolated using a 32-channel probe (A1x32-Poly3–10 mm-50–177-OA32LP, NeuroNexus). A micromanipulator (MPC-200, Sutter) was used to insert the probe perpendicular to the OB surface and lowered to the correct recording depth (Davidson and Katz, 2007). Neural activity was then amplified, digitized, and recorded (32-Channel Recording System, TDT). Principal component analysis and spike sorting were performed using TDT software. A 3-step sorting interface (Training, Classification, and Sorting) started with an initial training period to collect and compute candidate waveforms. A Bayesian algorithm was then applied to classify all subsequent data into each waveform class. After manual observation to ensure adequately classified waveforms, sort codes were applied to recorded data in real time. Any subsequent data analysis was performed using custom MATLAB script.

### Odor delivery for in vivo electrophysiology recordings

We used the following odors (Sigma): Isoamyl Acetate, 1-pentanol, 2-methypyrazine, octanoic acid, allyl sulfide, menthyl acetate, 1-hexanol, propionic acid, benzaldehyde, 2-ethylphenol, 1-octanol, valeraldehyde, benzene, P-cymene, P-toyl acetate, anisole, anisaldehyde, cyclobutanecarboxylic acid, gamma-nonalactone, heptanolic acid, (+)-limonene, alpha-phellandrene, (+)-terpinen-4-ol, eucalyptol, (+) carvone, menthone, methyl acetate, propyl acetate, tert-butyl acetate, butyl methyl ether, hexane, 2-hexanone, 3-heptanone, eugenol, a-Ionone. During recordings, positive pressure was applied to undiluted odor headspace using a microinjection dispenser (Picospritzer II, Parker) and the odorized air was diluted in 0.6 L/min free flowing O_2_ controlled by flowmeter (Acrylic In-Line Flow Meter, VWR). Odor presentation is controlled using liquid-dispensing robot (F5200, I&J Fisnar).

### Calculations for odor concentrations

The theoretical headspace saturation concentration was calculated for each odorant using the equation: ppm = vapor pressure (mmHg)/760 mmHg × 10^6^. We then calculated the presenting concentration by the equation ppm = (headspace saturation concentration) * (headspace volume * total presentation volume^−1^).

### Animal preparation for in vivo two-photon imaging

We used both adult male and female mice (age: 8+ weeks) of the *Crh*-Cre; *Vgat*^*flox/flox*^; *Thy1-GCaMP6F* and *Vgat*^*flox/flox*^; *Thy1-GCaMP6F* strains. Mice were initially anesthetized with 3% Isoflurane and maintained at 1.5–2% for the duration of the surgery. Body temp during surgery was maintained between 36.5 °C and 37.5 °C using a homeothermic blanket system (Harvard Instruments). In the surgical procedure, bupivicane (0.05 cc, 0.5%, Marcain) was applied subcutaneously under the scalp, and the skin over the skull was resected, and underlying fascia was removed. Wound margins were sealed with a thin layer of tissue adhesive (VetBond, 3M). A head-post metal bar was secured to the skull using cement, and the skull was held stationary on a small platform using an attached head bar. Using a surgical drill, a ~3 mm craniotomy was made over the olfactory bulb of both hemispheres, and exposed cortex was washed with ACSF (125 mM NaCl, 5 mM KCl, 10 mM glucose, 10 mM HEPES, 2 mM CaCl_2_, 2 mM MgSO_4_). Dura was removed, and the cortical window was then sealed with a 4 mm coverslip (Warner Instruments) using tissue adhesive. After surgery, animals were transferred to a cylindrical treadmill and given at least 30 min to fully recover from the effects of anesthesia and acclimate to head fixation.

### Two-photon imaging

We recorded calcium traces using a two-photon resonant microscope (Thorlabs) equipped with a Chameleon Ti-Sapphire laser (Coherent) tuned at 920 nm through a ×25, 1.1 NA Nikon objective. The average power output of the objective was kept at less than 150 mW, and calcium activity was typically sampled at ~12 Hz. We recorded data from depths of 100–450 μm below the cortical surface.

### Odor delivery for two-photon imaging

Different concentrations of odors were diluted v/v in mineral oil to reach presentation concentration of 100 ppb, a level that is above TC/MC detection threshold, but below saturation for most odors previously tested^29^. For all the two-photo imaging experiments we used the following odors: Odor Set 1 (ethyl acetate, butyl acetate, (+) Limonene, (−) Limonene, 2-heptanone, 2-hexanone, and 3-heptanone) and Odor Set 2 (1-butanol, 1-pentanol, 1-hexanol, 1-heptanol, 1-octanol, isoamyl acetate, and isoamyl butyrate). Odorants within each odor set was presented as both individuals and as 2-odor mixtures with every other odor within the set. To deliver odorants we used a custom-build automated 8-channel olfactometer in which the flow of each odor vial was coupled to a solenoid valve (Lee Company). The experiment was controlled by a custom state system written in LabView (National Instruments). Odorants were presented pseudo-randomly for 4 s, with 20 s of air flow in between. Each odor was presented at minimum 5 times.

### Preprocessing of calcium data

All analysis was performed in MATLAB (Mathworks), and the data processing chain for this and subsequent analysis relied on the DataJoint library for MATLAB (https://datajoint.github.io/). We compensated for motion artifacts in the horizontal plane, and cell detection was performed manually. TC bodies were localized at >100 μm deep (below the glomeruli and within the EPL) and MCs were identified in the mitral cell layer ~300 μm deep. Calcium traces were averaged from all pixels in segmented cells, and were normalized to ΔF/F. To estimate the responses to olfactory cues, we averaged the calcium activity during the odor presentation, and subtracted from the average calcium activity during the 2s prior to odor onset, unless otherwise stated.

### Analysis of calcium data

Excitatory responses are significantly higher than the 2s baseline activity before odor onset and suppressive responses are significantly lower (*p* < 0.05, one-tailed *t*-test). Tuning width: To estimate the tuning width of glomeruli and TC/MC bodies, we sorted responses to all odors after subtracting the baseline and fitted an exponential function $f\left( r \right) = ae^{br}$. We then used the full width half max of the exponential fit as a measure of the tuning width. Reliability: The reliability of each unit was defined as the % variance explained between the single trial responses and the average across all trials. It is estimated as the true variance, the variance of the average response across trials, divided by the observed variance, the total variance across all responses. If all responses to an odor are highly reliable then the variance across the average will be small, and thus the reliability measure closer to 100%. Regression analysis: We used a linear regression model to predict the responses to the odor mixtures from the responses to the two odors when presented individually. The calcium traces were binned at 200 ms, and a response vector of 12s total (2s pre-odor, 4s during odor presentation and 6s post-odor) was used for each odor. We implemented 2-fold cross-validation method. The explained variance of the model was computed as the average squared correlation coefficient between the predicted response from the single odor presentations, and the actual response to the mixture that were left out. The statistics were done over cells after averaging across odors and we report the median and 90% bootstrap confidence intervals with 5000 resamples unless otherwise stated. Signal correlation analysis: Signal correlations were measured as the Pearson correlation between tuning functions which were estimated as the median across trials of the average activity in the first two seconds after the odor onset.

### TC model

For all three TC models, we used as an input the average tuning functions of 300 tuned TCs recorded from Vgat KO animals. We subtracted the baseline from each tuning curve and randomized the odor preferences across 28 unique odors that were presented to simulate a uniform selectivity across the population. For Model 1, each response $x_i$ was transformed by $f(x_i) = \gamma x_i$. For the divisive normalization models (Models 2, 3), each response *x*_*i*_ was transformed by $f(x_i) = \gamma \frac{{x_i^2}}{{\sigma + \beta \mathop {\sum }\nolimits_j w_{ij}x_j}}$. For Model 2 the weights were fixed to 1. For Model 3 the weights w_ij_ were estimated as follows: we computed the correlation matrix across all 300 cells, then raised to the fourth power after normalizing each row between 0 and 1. That provided a good proxy of the tuning similarity between cells. Randomizing the indices of the correlation matrix provided similar results to Model 2 (Model 2*, Supplementary Fig. 8). We estimated the constants *γ*, *σ*, and *β* separately for each cell, and each model by fitting the output of $f(x_i)$ to the median tuning function across 199 tuned TCs in control animals. We implemented leave-one-out cross-validation method in which we evaluated the fit of the model to the response of one of the odors that was held out during fitting. As an output of all three models, we used these responses as rates to draw spike counts from a Poisson distribution (200 trials). We estimated the goodness of fit for each model as the root mean square error (RMSE) of the fit to the median TC tuning function from the control animals (Supplementary Fig. 8). The tuning width of each cell in all three models was estimated as the full width half max of the exponential fit as previously described. To assess the nonlinearity of the responses of the models we used as mixture inputs the sum of the tuning functions with a circular shifted version of the tuning function elements. We then computed the ratio between the model responses to the simulated mixture inputs and the sum of the mixture components. To quantify the cluster separation in the two-dimensional scatter plot in Fig. 8b we computed the average mahalanobis distance between each unit of the model to all of the control data points.

### Viral injection

Retrograde serotype Adeno-associated viruses (AAV) encoding an *Ef1a-H2b-mRuby2* was cloned and packaged in house. 690 nl Retro-AAV was injected into the anterior piriform cortex of both *Crh*-Cre; *Vgat Flox/Flox*; *Thy1-Gcamp6F* and *Vgat Flox/Flox*; *Thy1-Gcamp6F* mice using glass injection pipettes and a Nanoject II (Drummond Scientific Company, Broomall, PA) at a rate of 69 nL s^−1^ at 30 s intervals. Animals were imaged 2 weeks after injection.

### Reporting summary

Further information on research design is available in the Nature Research Reporting Summary linked to this article.

## Supplementary information

Supplementary Information

Reporting Summary

## Data Availability

The data that support the findings of this study are available from the corresponding author upon reasonable request.

## Code availability

The code that support the findings of this study are available from the corresponding author upon reasonable request.
