# Supplementary material for: Target specific functions of EPL interneurons in olfactory circuits
Source: Nat Commun. 2019 Jul 29;10:3369. doi: 10.1038/s41467-019-11354-y (PMC6662826; doi:10.1038/s41467-019-11354-y)
Supplement: Supplementary file 1 — Supplementary Information [file 41467_2019_11354_MOESM1_ESM.pdf]

SUPPLEMENTARY INFORMATION PDF

Liu and Froudarakis et al

Liu and Froudarakis et al., Supplementary Table 1

Odorants and functional groups for MEA recordings

Odor	Number	Functional Group	Odor	Number	Functional Group
Isoamyl Acetate	1	Ester	Gamma-nonalactone	19	Cyclic Ester
1-Pentanol	2	Alcohol	Heptanoic Acid	20	Carboxylic Acid
2-Methypyrazine	3	Nitrogen	(R)-(+)-Limonene	21	Cyclic Alkene
Octanoic Acid	4	Carboxylic Acid	Alpha-phellandrene	22	Cyclic Alkene
Allyl Sulfide	5	Sulfide	(+)-Terpinen-4-ol	23	Cyclic Alcohol
Menthyl Acetate	6	Cyclic Ester	Eucalyptol	24	Cyclic Oxygen
1-Hexanol	7	Alcohol	(+) Carvone	25	Cyclic Ketone
Propionic Acid	8	Carboxylic Acid	Menthone	26	Cyclic Ketone
Benzaldehyde	9	Aromatic Aldehyde	Methyl Acetate	27	Ester
2-Ethylphenol	10	Aromatic Alcohol	Propyl Acetate	28	Ester
1-Octanol	11	Alcohol	Tert-Butyl Acetate	29	Ester
Valeraldehyde	12	Aldehyde	Butyl Methyl Ether	30	Ester
Benzene	13	Aromatic Carbohydrate	Hexane	31	Hydrocarbon
P-Cymene	14	Aromatic Carbohydrate	2-Hexanone	32	Ketone
P-Toyl Acetate	15	Aromatic Ester	3-Heptanone	33	Ketone
Anisole	16	Aromatic Ether	Eugenol	34	Aromatic alcohol & Ether
Anisaldehyde	17	Aromatic Ketone	a-Ionone	35	Cyclic Ketone
Cyclobutane Carboxylic Acid	18	Carboxylic Acid	Mineral Oil Control	36	

Liu and Froudarakis et al., Supplementary Table 2

a

Odor Concentrations for MEA recordings

Odor	Calculated Presenting Concentration (PPM)	Odor	Calculated Presenting Concentration (PPM)
Isoamyl Acetate	52.63	Gamma-nonalactone	0.1553
1-Pentanol	586.8	Heptanoic Acid	0.1408
2-Methypyrazine	127.4	(R)-(+)-Limonene	26.32
Octanoic Acid	0.04882	Alpha-phellandrene	24.42
Allyl Sulfide	121.3	(+)-Terpinen-4-ol	0.6316
Menthyl Acetate	2276	Eucalyptol	25
1-Hexanol	104.1	(+) Carvone	2.105
Propionic Acid	46.45	Menthone	6.579
Benzaldehyde	13.16	Methyl Acetate	2276
2-Ethylphenol	2.013	Propyl Acetate	472.4
1-Octanol	1.842	Tert-Butyl Acetate	618.4
Valeraldehyde	342.1	Butyl Methyl Ether	8129
Benzene	1118	Hexane	2013
P-Cymene	19.74	2-Hexanone	128.3
P-Toyl Acetate	2.105	3-Heptanone	52.63
Anisole	46.58	Eugenol	0.2908
Anisaldehyde	0.3276	a-Ionone	0.1842
Cyclobutane Carboxylic Acid	2.368	Mineral Oil Control	

b

Odor Concentrations for 2-Photon Imaging

Odor Set 1	Calculated Presenting Concentration (PPB)	Odor Set 2	Calculated Presenting Concentration (PPB)
Ethyl Acetate	100	1-Butanol	100
Butyl Acetate	100	1-Pentanol	100
(+) Limonene	100	1-Hexanol	100
(-) Limonene	100	1-Heptanol	100
2-Heptanone	100	1-Octanol	100
2-Hexanone	100	Isoamyl Acetate	100
3-Heptanone	100	Isoamyl Butyrate	100

Liu and Froudarakis et al., Supplementary Figure 1

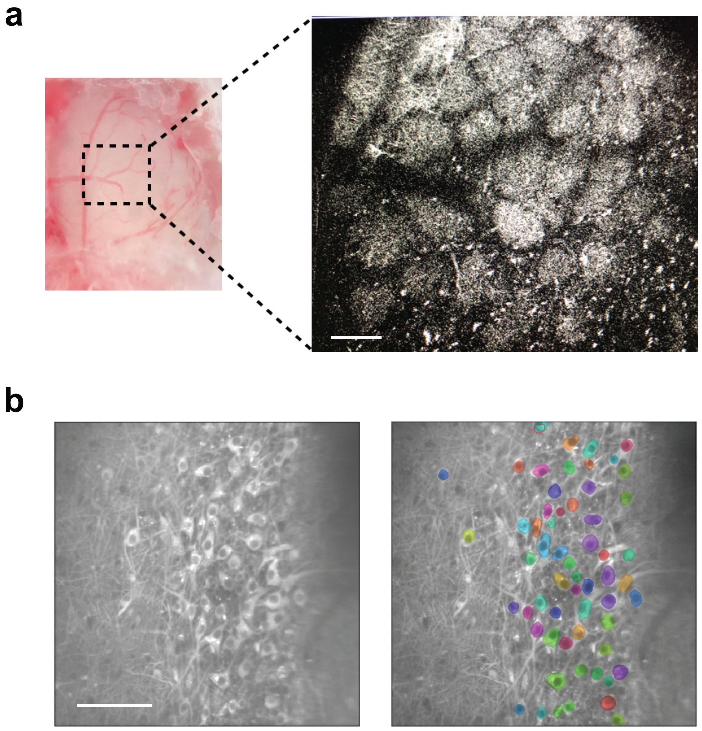

(a) OB window (left) and the dotted box imaged with in vivo 2-photon microscopy (right). Blood vessel patterns are utilized to image at center of OB. (b) 2-photon image of MCs before manual segmentation of masks (left) and after manual segmentation of masks (right). Scale bar 100um.

Liu and Froudarakis et al., Supplementary Figure 2

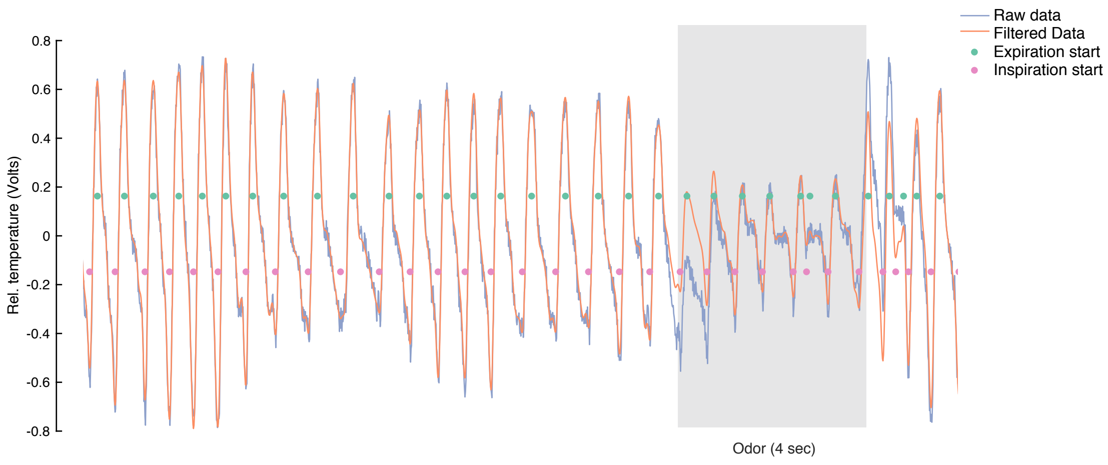

Processing of air flow temperature recordings outside of mice nostrils. Grey is the unfiltered voltage, Red is the filtered, normalized data. Peaks occur at start of expiration and troughs occur at start of inspiration.

Liu and Froudarakis et al., Supplementary Figure 3

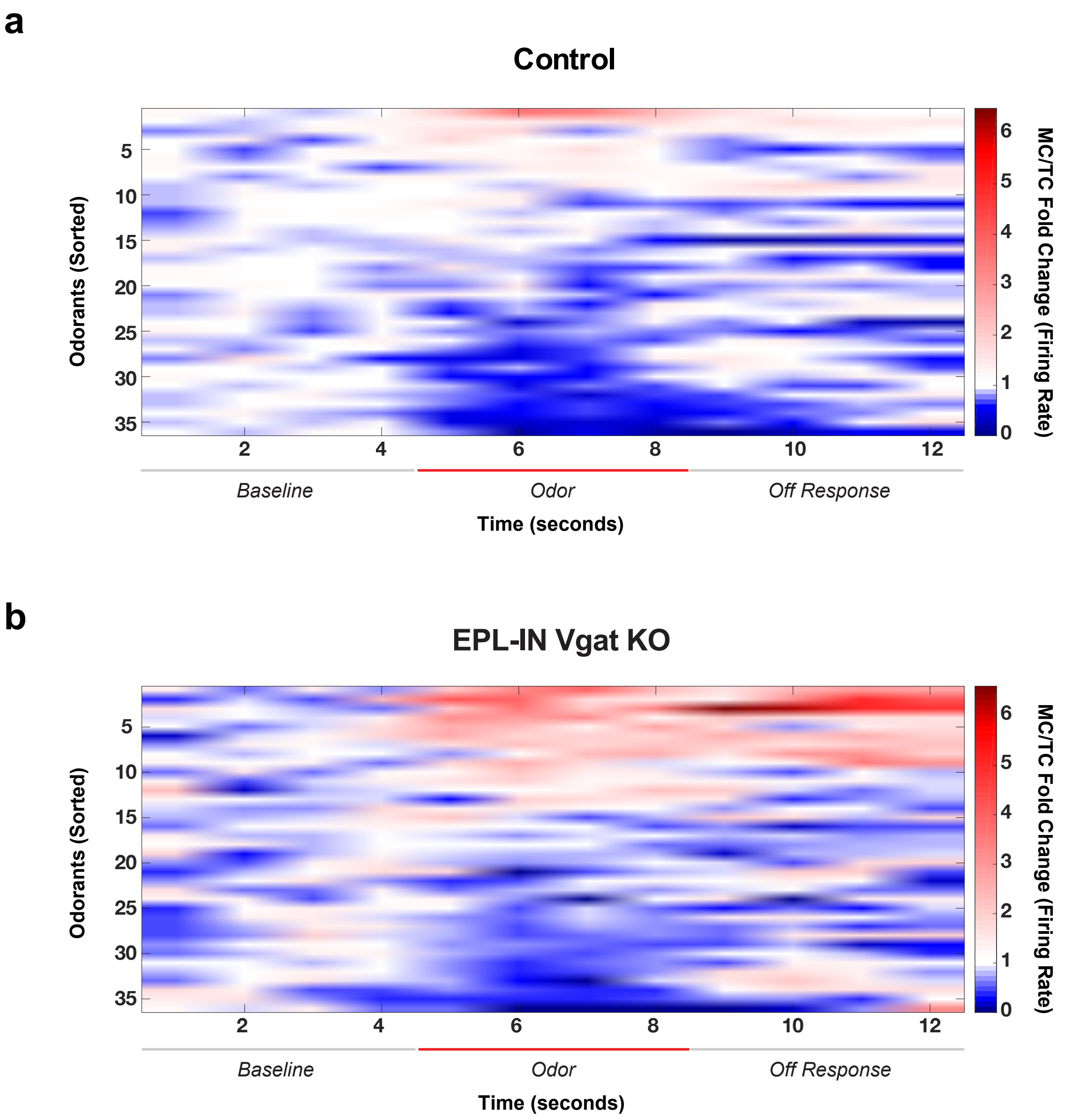

Single cell odor response profile including the odor off response from TC or MC of (a) control and (b) EPL-IN Vgat knockout mice

Liu and Froudarakis et al., Supplementary Figure 4

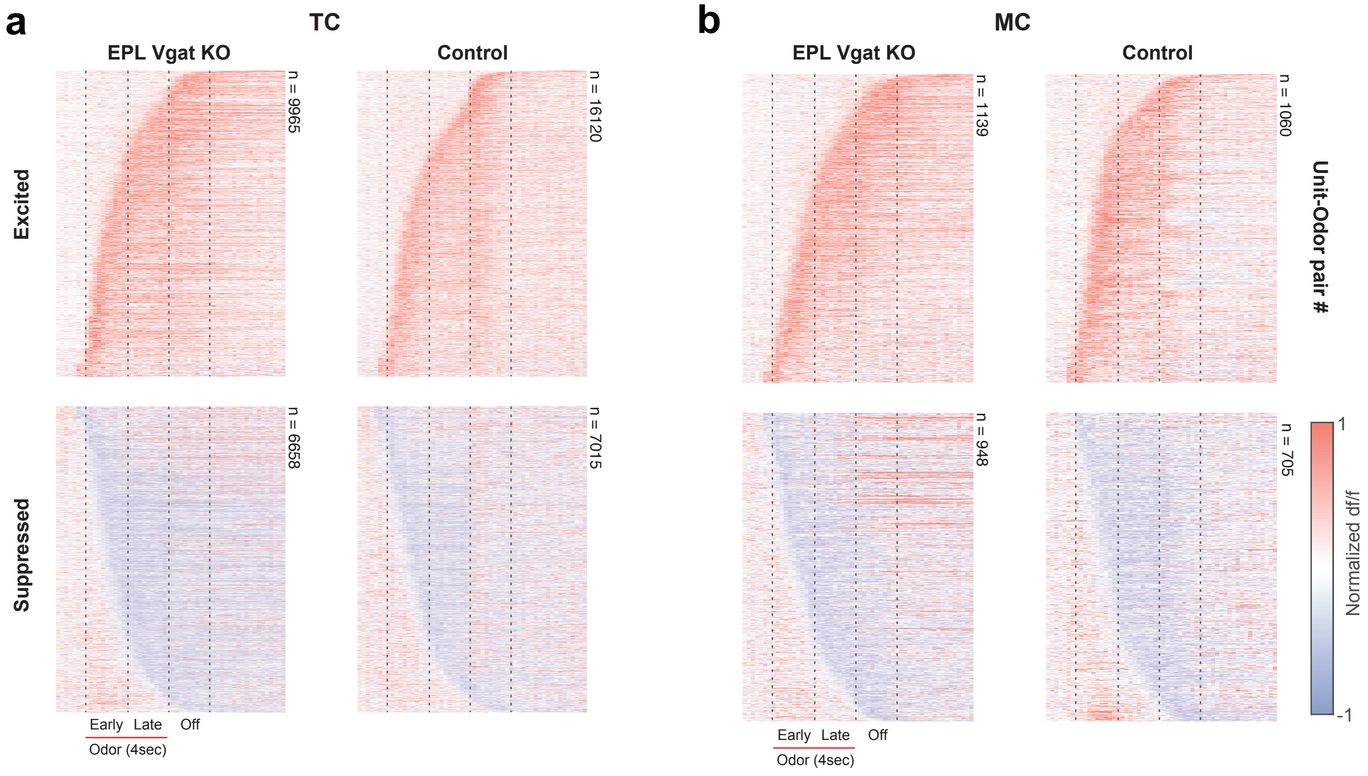

Calcium responses to odors across all significant unit-odor pairs sorted by latency for excited (top) and suppressed (bottom) units between EPL Vgat KO (left) and control (right) animals for two classes of cells: (a) TC (left two columns), and (b) MC (right two columns). Time is displayed on the horizontal axis, and unique unit-odor pairs on the vertical axis. Calcium responses were normalized by either their maximum (excited) or minimum (suppressed) value. Responses are excitatory if the average response during 2s of odor presentation or 2s following odor presentation were significantly higher than the average 2s baseline before the onset of the stimulus, and suppressive if it was significantly lower ($p<0.05$, one-tailed t-test). Dotted lines denote transition between different phases of odor presentation.

Liu and Froudarakis et al., Supplementary Figure 5

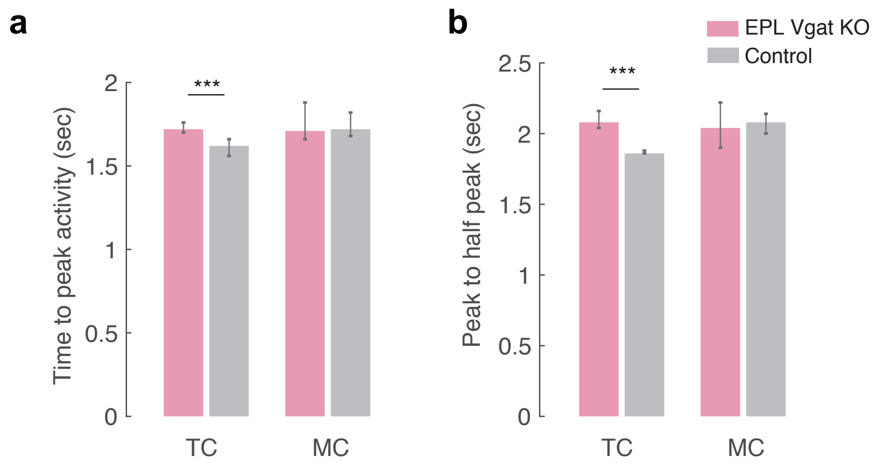

Temporal dynamics of odor responses that were significantly excited during the first 2 seconds of the odor presentation between EPL Vgat KO and Control. (a) Time to peak activity after odor onset and (b) Time from peak activity down to half peak. The number of cells for EPL Vgat KO animals: TC = 4991 and MC = 460, for control animals: TC = 6475, MC = 519. ***$p < 0.001$, Wilcoxon rank sum test. Error bars show SEM.

Liu and Froudarakis et al., Supplementary Figure 6

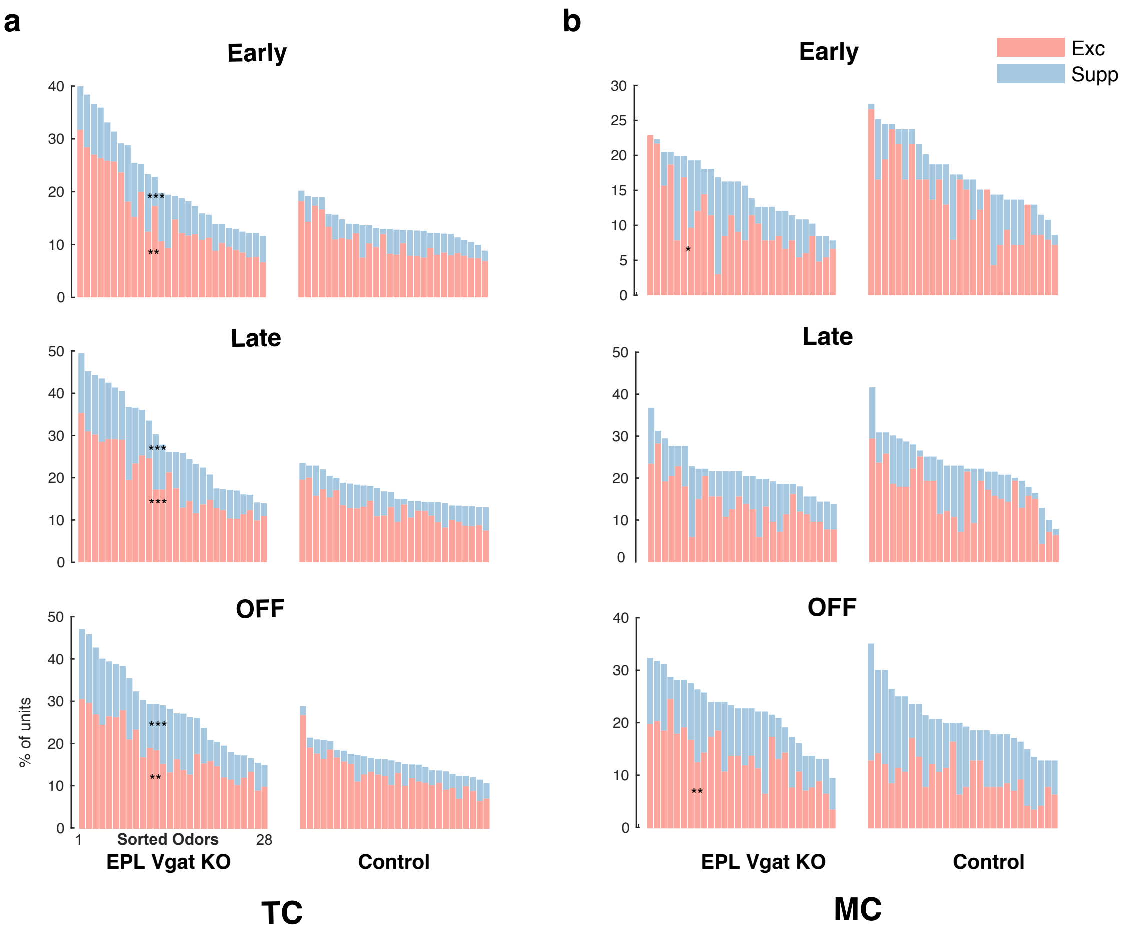

Histograms represent the cellular responses to each odor that were significantly excited or suppressed for (a) TC and (b) MC between EPL Vgat KO and Controls. The fraction of unit-odor pairs is shown on the Y axis, and average response during odor presentation on the x axis. Responses divided into Early (first 2s of odor), Late (last 2s of odor), and Off (2s after odor stops). (c) Histograms showing the fraction of excited, suppressed, and unresponsive cell-odor pairs across all recordings. The number of cells for EPL Vgat KO animals: TC = 1214, MC = 166, for control animals: TC = 2396, MC = 139. *p < 0.05, **p < 0.01, ***p < 0.001, Wilcoxon rank sum test.

Liu and Froudarakis et al., Supplementary Figure. 7

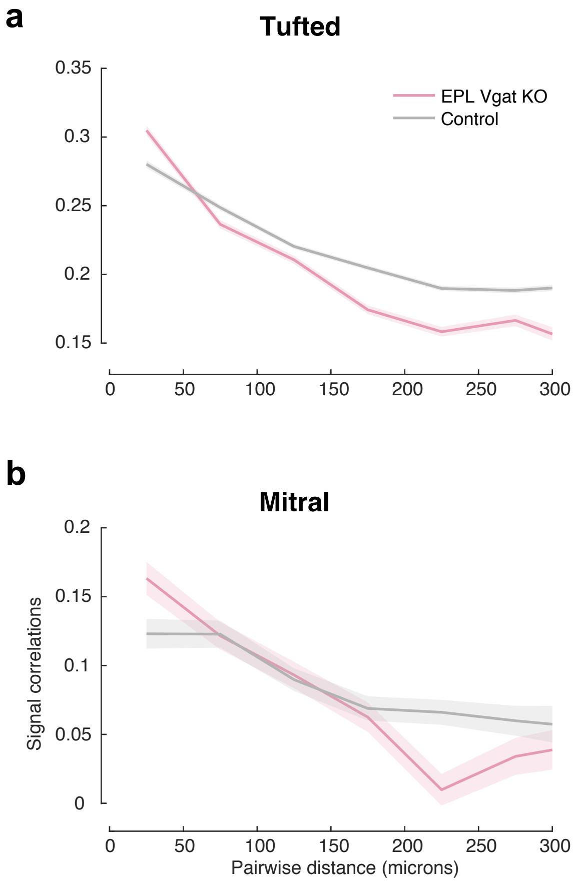

Tuning similarity as a function of spatial separation. The tuning similarity, estimated as the signal correlations between the cells, is plotted against the Euclidean horizontal distance between the cells from the same scan for TCs (top) and MCs (bottom) for both experimental (magenta) and control (grey) animals). The number of cell pairs for EPL Vgat KO animals: TC = 10719, MC = 594, for control animals: TC = 12783, MC = 504. Shaded area around the mean correlation represents SEM over cells.

Liu and Froudarakis et al., Supplementary Figure 8

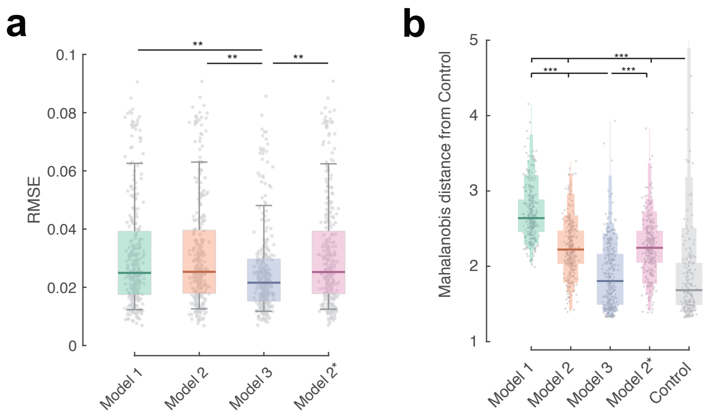

(a) Goodness of fit estimated as the root mean square error (RMSE) for each of the 4 models. lower = more accurate model. Box plots are computed across all units (n=300) - bounds of the box spans from 25 to 75% percentile, center line represents median, and whiskers visualize 10 and 90% of the data points. (b) Average mahalanobis distances as in Figure 8c between each of the cells of model/control performance and the control data. Lines indicate the median of the distribution, and the variable bars indicate the 75, 90, 95 and 100 percentiles. Dots represent the d' for each unit (n=300 for all four models). **p < 0.001, ***p < 10-24, Wilcoxon rank sum test.
