# Supplementary material for: Target specific functions of EPL interneurons in olfactory circuits
Source: Nat Commun. 2019 Jul 29;10:3369. doi: 10.1038/s41467-019-11354-y (PMC6662826; doi:10.1038/s41467-019-11354-y)
Supplement: Supplementary file 2 — Reporting Summary [file 41467_2019_11354_MOESM2_ESM.pdf]

nature research

Corresponding author(s): Benjamin R Arenkiel

Last updated by author(s): Jun 25, 2019

Reporting Summary

Nature Research wishes to improve the reproducibility of the work that we publish. This form provides structure for consistency and transparency in reporting. For further information on Nature Research policies, seeAuthors & Referees and theEditorial Policy Checklist .

Statistics

For all statistical analyses, confirm that the following items are present in the figure legend, table legend, main text, or Methods section.

n/a	Confirmed	
☐	☒	The exact sample size (*n*) for each experimental group/condition, given as a discrete number and unit of measurement
☐	☒	A statement on whether measurements were taken from distinct samples or whether the same sample was measured repeatedly
☐	☒	The statistical test(s) used AND whether they are one- or two-sided<br>*Only common tests should be described solely by name; describe more complex techniques in the Methods section.*
☐	☒	A description of all covariates tested
☐	☒	A description of any assumptions or corrections, such as tests of normality and adjustment for multiple comparisons
☐	☒	A full description of the statistical parameters including central tendency (e.g. means) or other basic estimates (e.g. regression coefficient) AND variation (e.g. standard deviation) or associated estimates of uncertainty (e.g. confidence intervals)
☐	☒	For null hypothesis testing, the test statistic (e.g. F, t, r) with confidence intervals, effect sizes, degrees of freedom and P value noted<br>*Give P values as exact values whenever suitable.*
☒	☐	For Bayesian analysis, information on the choice of priors and Markov chain Monte Carlo settings
☒	☐	For hierarchical and complex designs, identification of the appropriate level for tests and full reporting of outcomes
☐	☒	Estimates of effect sizes (e.g. Cohen's *d*, Pearson's *r*), indicating how they were calculated

Our web collection on statistics for biologists contains articles on many of the points above.

Software and code

Policy information about availability of computer code

Data collection	To collect the two-photon data we used ScanImage 5.2 from Vidrio Technologies. The control of the hardware for odor presentation was done with custom written programs in Labview 2016 from National instruments. To collect the slice electrophysiology data we used Clampex 10.6.2.2 software from Molecular Devices. In vivo electrophysiology recordings were performed using Synapse software from Tucker Davis Technology. Wide-field GCaMP imaging was performed using LAS AF software from Leica.
Data analysis	All data analysis for two-photon data was done with custom written code in Matlab 2017 from Mathworks. The code will be available upon request. Analysis of slice electrophysiology data was done using MiniAnalysis (Synaptosoft Inc, Fort Lee, NJ) and Clampfit 10.7.0.3 (Molecular Devices) software. Processing of in vivo electrophysiology data was performed using Synapse software from Tucker Davis Technology. Wide-field GCaMP data was processed into odor maps using custom Fiji script. The code will be available upon request.

For manuscripts utilizing custom algorithms or software that are central to the research but not yet described in published literature, software must be made available to editors/reviewers. We strongly encourage code deposition in a community repository (e.g. GitHub). See the Nature Research guidelines for submitting code & software for further information.

Data

Policy information about availability of data

All manuscripts must include a data availability statement. This statement should provide the following information, where applicable:

- Accession codes, unique identifiers, or web links for publicly available datasets
- A list of figures that have associated raw data
- A description of any restrictions on data availability

The data that support the findings of this study are available from the corresponding author upon reasonable request.

Field-specific reporting

Please select the one below that is the best fit for your research. If you are not sure, read the appropriate sections before making your selection.

- [x] Life sciences
- [ ] Behavioural & social sciences
- [ ] Ecological, evolutionary & environmental sciences

For a reference copy of the document with all sections, see nature.com/documents/nr-reporting-summary-flat.pdf

Life sciences study design

All studies must disclose on these points even when the disclosure is negative.

Sample size	Sample sizes were determined based on prior experiments which utilized the same techniques. For juxtacellular electrophysiological recordings, we sampled enough cells to achieve at least 1 cell for every odor presented. This allowed for an adequate odor response curve to be generated. For in vivo 2-photon imaging, we were able to gather hundreds to thousands of odor-glomeruli/cell pairs, giving us sufficient power in this experiment.
Data exclusions	N/A
Replication	Each experiment contain multiple animals from different litters, and each animal was imaged/recorded on different days. We found that all of our results can adequately be replicated.
Randomization	Both control and experimental animals were subjected to the same experimental conditions. The only difference is that experimental animals have a different genotype than the control.
Blinding	Cell density analysis was performed by participants who were blinded to the animal genotypes. Both electrophysiological and 2-photon recordings were performed and analyzed by blinded individuals.

Reporting for specific materials, systems and methods

We require information from authors about some types of materials, experimental systems and methods used in many studies. Here, indicate whether each material, system or method listed is relevant to your study. If you are not sure if a list item applies to your research, read the appropriate section before selecting a response.

Materials & experimental systems

n/a	Involved in the study	
[ ]	[x]	Antibodies
[x]	[ ]	Eukaryotic cell lines
[x]	[ ]	Palaeontology
[ ]	[x]	Animals and other organisms
[x]	[ ]	Human research participants
[x]	[ ]	Clinical data

Methods

n/a	Involved in the study	
[x]	[ ]	ChIP-seq
[x]	[ ]	Flow cytometry
[x]	[ ]	MRI-based neuroimaging

Antibodies

Antibodies used	anti-Calretinin 1:1500 (MAB1568, Chemicon), mouse anti-Parvalbumin 1:3000 (MAB1572, Millipore), rabbit anti-Tyrosine Hydroxylase 1:2000 (Ab152, Chemicon), or rabbit anti-Tbx21 1:500 (gift from Mitsui Lab).
Validation	Calretinin References: http://www.emdmillipore.com/US/en/product/Anti-Calretinin-Antibody-clone-6B8.2,MM_NF-MAB1568#anchor_REF<br><br>anti-Parvalbumin references: http://www.emdmillipore.com/US/en/product/Anti-Parvalbumin-Antibody,MM_NF-MAB1572#anchor_REF<br><br>anti-Tyrosine Hydroxylase references: http://www.emdmillipore.com/US/en/product/Anti-Tyrosine-Hydroxylase-Antibody,MM_NF-AB152#anchor_REF<br><br>anti-Tbx21 references: https://www.ncbi.nlm.nih.gov/pmc/articles/PMC4362939/

Animals and other organisms

Policy information about studies involving animals; ARRIVE guidelines recommended for reporting animal research

Laboratory animals	Crh-Cre+/- (Crhtm1(cre)Zjh), Vgatflox (Slc32a1tm1Lowl), ROSA26 Lox-stop-Lox ChR2-EYFP (GT(ROSA)26Sortm32.1(CAG-COP4*H134R/EYFP)Hze/J), and Thy1-GCaMP6f (Tg(Thy1-GCaMP6f)GP5.11Dkim)) mice were obtained from the Jackson Laboratories and bred under standard breeding schemes to achieve the desired strains. Study includes both male and female for each experiment, adult mice age 8+ weeks.
Wild animals	No wild animals were used in this study
Field-collected samples	No field collected samples were used in this study
Ethics oversight	All mice work performed in this study was approved by Baylor College of Medicine's IRB under multiple study protocols.

Note that full information on the approval of the study protocol must also be provided in the manuscript.
